# Early Life Social Isolation Dysregulates Social Reward Processing, BDNF Signaling, and Intracellular Vesicular Sorting in the Nucleus Accumbens of Male and Female Rats

**DOI:** 10.1111/jnc.70181

**Published:** 2025-08-05

**Authors:** M. Di Trapano, V. Buzzelli, B. Rizzi, F. Mottarlini, S. Schiavi, R. Ciccocioppo, L. Fattore, P. Romualdi, F. Fumagalli, V. Trezza, L. Caffino, A. Manduca

**Affiliations:** ^1^ Department of Science, Section of Biomedical Sciences and Technologies Roma Tre University Rome Italy; ^2^ Center for Neuroscience University of Camerino Camerino Italy; ^3^ Department of Pharmacological and Biomolecular Sciences ‘Rodolfo Paoletti’ University of Milan Milan Italy; ^4^ School of Pharmacy, Center for Neuroscience, Pharmacology Unit University of Camerino Camerino Italy; ^5^ Research National Council (CNR) Institute of Neuroscience‐Cagliari National Research Council Cagliari Italy; ^6^ Department of Pharmacy and Biotechnology University of Bologna Bologna Italy; ^7^ Neuroendocrinology, Metabolism and Neuropharmacology Unit IRCCS Fondazione Santa Lucia Rome Italy; ^8^ Department of Physiology and Pharmacology Sapienza University of Rome Rome Italy

**Keywords:** early‐life isolation, endocytic‐recycling pathway, nucleus accumbens, sex difference, social reward

## Abstract

Early‐life social deprivation negatively impacts brain development and behavior, increasing susceptibility to neuropsychiatric disorders. In social species such as rats, interactions with the mother and conspecifics are crucial for offspring survival and proper neurobehavioral maturation. However, the mechanisms underlying sex‐dependent vulnerability to early‐life social stressors, such as social isolation, remain unclear. This study aimed to (i) investigate the effects of early‐life social isolation (ESI) on social and depressive‐like behaviors in female and male rats during adolescence and adulthood and (ii) explore the molecular mechanisms involved, focusing on the BDNF system in the nucleus accumbens (NAc), a key brain region for social behavior and reward processing. To this aim, we implemented an ESI protocol involving brief periods of repeated social isolation from postnatal day (PND) 14–21 to mimic an adverse early social environment, and then we tested female and male rats across development (i.e., during adolescence and adulthood). Our findings revealed that ESI impaired social reward processing in male rats, whereas general social and depressive‐like behaviors remained unaffected in both sexes. These behavioral deficits were accompanied by sex‐dependent effects on the BDNF/TrkB signaling pathway in the NAc. Specifically, males exhibited a persistent ESI‐induced downregulation of BDNF signaling paralleled by alterations in endocytic‐recycling mechanisms mediated by Rab5‐Rab11, suggesting increased TrkB sorting and reduced neuroplasticity. Conversely, females showed increased BDNF signaling and enhanced early endosome‐recycling mechanisms. These results suggest that male and female rats rely on distinct neurobiological mechanisms to modulate reward processing in response to early‐life stress. Overall, our study highlights sex‐specific, long‐lasting effects of ESI on social reward processing and molecular pathways, providing insight into differential susceptibility to social adversity.

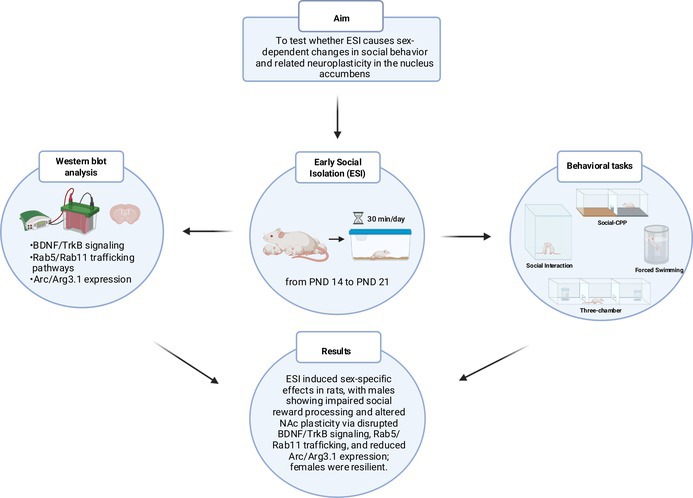

## Graphical abstract

Early‐life social isolation (ESI) impacts brain development and may increase vulnerability to neuropsychiatric disorders. In this study, brief ESI (PND 14–21) induced sex‐specific, long‐lasting effects on social reward behavior and neuroplasticity in rats. Males showed deficits in social reward processing across development, associated with reduced BDNF/TrkB signaling and altered Rab5‐Rab11 endosomal trafficking in the nucleus accumbens. In contrast, females were resilient, displaying enhanced BDNF signaling and preserved trafficking mechanisms. These findings suggest that males and females engage distinct molecular pathways in response to early social stress, revealing a critical role for the BDNF/TrkBRab axis in sex‐dependent vulnerability.

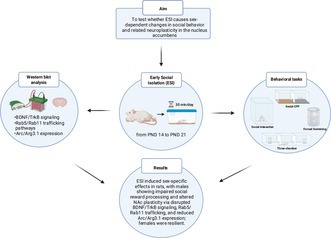

**Abbreviations**
AKT: protein kinase B
Arc: activity‐regulated cytoskeletal associated protein
BDNF: brain‐derived neurotrophic factor
CS: conditioned stimulus
CTRL: control
ESI: early social isolation
MAPK: mitogen‐activated protein kinase
NAc: nucleus accumbens
PI3K: phosphatidylinositol 3‐kinase
PND: post‐natal day
sCPP: socially‐induced conditioned place preference
TBS: tris‐buffered saline
TrkB: tyrosine receptor kinase B

## 1 Introduction

Mammals lacking appropriate social stimuli during development suffer from profound physiological and behavioral complications (Orben et al. 2020). In humans, negative social experiences, such as early‐life social isolation, increase vulnerability to psychiatric disorders later in life, including (but not limited to) depression, anxiety disorders, schizophrenia, and substance abuse (Chandan et al. 2019; Brandt et al. 2022). In social species such as rats, interactions with the mother and conspecifics are crucial for offspring survival, as well as for ensuring proper neurobehavioral maturation. Disruptions in these relationships may result in profound neuroendocrine, neurochemical, and behavioral alterations that may persist across development (Manduca et al. 2021; Keller et al. 2019; Cirulli et al. 2003; Kaplan et al. 2025). Repeated maternal separation is an early‐life social stressor in rodents involving brief daily separations of pups from their mother. These separations do not exceed the typical separation bouts occurring when the mother naturally leaves the nest and, therefore, are not considered a form of maternal deprivation (Weiss et al. 2001). The social isolation (single housing in a cage with clean bedding) of pups for 30 min/day before weaning from postnatal day (PND) 14–PND 21 (i.e., early social isolation, ESI), an age range comparable to childhood in humans (Chini and Hanganu‐Opatz 2021), is a mild aversive situation that induces subthreshold behavioral changes in the rat offspring. In fact, we recently demonstrated that brief periods of ESI alter sensorimotor gating and compulsive‐like behaviors (Bratzu et al. 2023), increase the sensitivity of female Marchigian Sardinian alcohol‐preferring rats to yohimbine‐induced alcohol seeking (Benvenuti et al. 2024), dysregulate the endocannabinoid and glucocorticoid receptor signaling (Rullo et al. 2023) and reorganize the molecular composition of the glutamatergic synapse in the prefrontal cortex of female rats (Mottarlini, Rizzi, et al. 2024). Additionally, mice subjected to this brief social isolation procedure during the third postnatal week displayed depressive‐like behaviors in adulthood, associated with epigenetic changes in various brain regions (Catale et al. 2020; Lo Iacono et al. 2015). However, little is known about how ESI during the third postnatal week results in sex‐specific behavioral and molecular vulnerability in the brain of developing rodent pups.

The nucleus accumbens (NAc) is a central component of the brain's reward circuitry, playing a central role in motivated behavior, including reward processing (Russo and Nestler 2013). Beyond its involvement in reward‐related behaviors, the NAc is essential for social adaptation, contributing to prosocial behaviors, the formation of emotional bonds between individuals (Walsh et al. 2023; Dolen et al. 2013; Waguespack et al. 2024; Shan et al. 2022), and for the establishment of social preference, a process driven by social reward (Li et al. 2025; Park et al. 2021). Consequently, NAc dysfunctions have been associated with psychiatric disorders characterized by social deficits, such as depression and autism spectrum disorders (Ding et al. 2022; Sato et al. 2023).

From a neurobiological perspective, the neurotrophin Brain‐derived Neurotrophic factor (BDNF) is a key molecule involved in reward‐related processes within the mesocorticolimbic pathway (Caffino et al. 2022; Caffino, Mottarlini, et al. 2020; Caffino, Giannotti, et al. 2019; Caffino, Mottarlini, et al. 2019; Li and Wolf 2015). Interestingly, BDNF‐induced neuroplastic mechanisms play a crucial role in shaping experience‐dependent social behaviors (Komori et al. 2024), and brain region‐specific dysregulations of the BDNF system have been widely used as markers of stress impact (Calabrese et al. 2015; Han et al. 2025) even in a condition of social deprivation (Berry et al. 2012). This highlights BDNF's essential role in guiding the brain's maturational trajectory following early‐life social isolation stress. BDNF exerts its neuroplastic action via binding to its high affinity receptor, TrkB, which upon autophosphorylation activates the MAPK and PI3K signaling pathways (Chao 2003). Interestingly, sustained activation of BDNF‐mediated intracellular signaling requires endocytic mechanisms, thus involving the activity of the Rab family of monomeric GTPase Rab5‐Rab11 (Zheng et al. 2008; Gonzalez‐Gutierrez et al. 2020). Dysfunction of this early‐recycling pathway impairs BDNF signaling, contributing to pathological conditions (Moya‐Alvarado et al. 2022).

Although the effects of social development in both normal and isolated environments have already been investigated (Xiong et al. 2023), the impact of ESI on social behavior and neuroplastic mechanisms within the reward system remains poorly understood. Furthermore, sex‐dependent vulnerability to social deficits and the underlying neurobiological mechanisms have received limited attention, likely due to the complexity of social isolation and translational gaps in findings from animal models. To address this gap, we applied an ESI protocol during the third postnatal week in male and female rats. We then explored the sex‐dependent consequences on social and depressive‐like behaviors and on the modulation of the BDNF system in the NAc. The effects of ESI were assessed at two developmental ages: adolescence (PND 35–45) and adulthood (PND 75–85) to evaluate the short‐ and long‐term impact of the early in‐life social manipulations. Understanding how early social stressors shape social behavior in male and female rodents across the lifespan and the underlying mechanisms has significant translational relevance for neuropsychiatric disorders.

## 2 Materials and Methods

### 2.1 Animals

Female Wistar rats (Charles River, Italy), weighing 250 ± 15 g, were mated overnight. The morning when spermatozoa were found was designated as gestational day 1. Pregnant rats were singly housed in Macrolon cages [40 (length) × 26 (width) × 20 (height) cm], under controlled conditions (temperature 20°C–21°C, 55%–65% relative humidity and 12/12 h light cycle with lights on at 07:00 h). Food and water were available ad libitum. Newborn litters found up to 17:00 h were considered to be born on that day [postnatal day (PND) 0]. On PND 1, the litters were culled to eight animals (four males and four females) to reduce the litter size‐induced variability in the growth and development of pups during the postnatal period (Agnish and Keller 1997). If dams had a smaller litter, pups were moved between litters of the same experimental group to ensure that all litters have the same size. In cases of surplus animals, they were either used to compensate for smaller litters within the same experimental group, or they were humanely euthanized following institutional ethical guidelines. Starting at PND 14, two males and two females per litter were arbitrarily subjected to the early social isolation (ESI) protocol as described below. On PND 21, pups were weaned and housed in groups of three of the same sex and same experimental condition but not littermates and then tested across development. Before starting the behavioral experiments, rats were handled daily for 5 min for 3 days by the same researchers who performed the experiments. The behavioral experiments were carried out on the male and female offspring during adolescence (PNDs 35–45) or adulthood (PNDs 75–85). Each animal was subjected to only one behavioral test at only one age (adolescence or adulthood) to avoid potential confounding effects due to repeated testing. This approach ensured the integrity and interpretability of each behavioral outcome while maintaining experimental control and minimizing the impact of testing history on the results. Another cohort of female and male offspring from each experimental group has been left undisturbed in their home cages following the end of the ESI protocol and then sacrificed at PND 35 or at PND 75, to evaluate the short‐ and long‐term impact of the early in life social manipulations on the BDNF system. Therefore, the behavioral and molecular experiments were performed in different animals to avoid potential influences of behavioral tasks on biochemical outcomes.

We used a total of 107 females (53 CTRL and 54 ESI) and 98 males (53 CTRL and 45 ESI). The exact sample size (*n*) for each experimental group/condition is indicated in the figure legends. The sample size was based on our previous experiments and power analysis performed with the software G* power. Potential outliers within each data set were calculated using Grubb's method. Scoring of the behavioral experiments was done in blind conditions using the Observer 3.0 software (Noldus, The Netherlands). Moreover, the operators for training/testing and scoring were different (i.e., the researchers who performed the test did not score the animals and vice versa). No randomization was performed to allocate subjects in the study. Subjects were arbitrarily allocated to the different behavioral or molecular experiments.

The experiments were approved by the Italian Ministry of Health (authorization n. 612/2020‐PR) and performed in agreement with the ARRIVE (Animals in Research: Reporting In Vivo Experiments) guidelines (Kilkenny et al. 2010), the guidelines of the Italian Ministry of Health (D.Lgs. 26/14) and the European Community Directive 2010/63/EU for the protection of animals used for scientific purposes.

**FIGURE 1:**
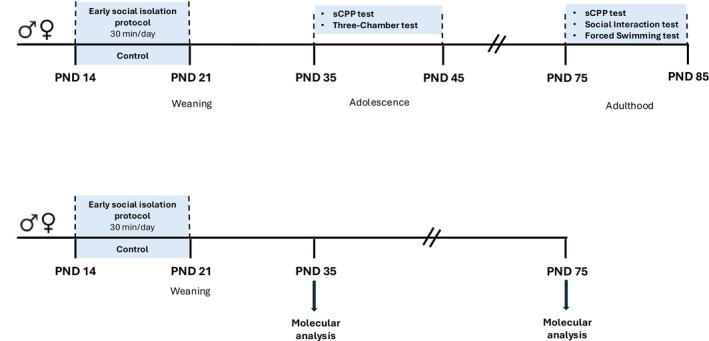
Timeline of the experiments. Sequence of the behavioral and molecular experiments performed in male and female rats following early social isolation (ESI) protocol. PND, post‐natal day; sCPP, socially‐induced conditioned place preference.

The timeline of the experiments is shown in Figure 1.

### 2.2 Early Social Isolation (ESI) Protocol

Early social isolation occurred during the light cycle, between 9:00 and 11:00 a.m. during PND 14–PND 21, as previously described (Benvenuti et al. 2024). Half of the pups from each nest were singly housed in a novel clean bedding cage for 30 min per day (i.e., each pup was isolated from both the dam and the siblings). After the 30 min separation time, they were returned to their home cages. Control pups were left undisturbed with their mothers in their home cages.

### 2.3 Behavioral Tests

#### 2.3.1 Socially‐Induced Conditioned Place Preference (sCPP)

The sCPP test was performed at adolescence and adulthood over four consecutive days as previously described (Schiavi et al. 2019; Wei et al. 2015). Briefly, rats were placed in an acrylic box [75 (length) × 35 (width) × 35 (height) cm], divided into two chambers by a clear acrylic wall with a small opening. Each chamber contained different types of autoclaved novel bedding (Sanyx Bio Ultra litter and Padovan Sandy Litter), which differed in texture and shade (white vs. dark‐brown). On Day 1, a 30‐min pre‐conditioning test was used to establish any innate preference for either of the two types of novel bedding. Individual rats with a strong preference for either type of bedding were excluded (typically, those that spent more than 1.5× time on one bedding over the other). The next day (Day 2), animals were assigned to a social cage (CS+) to be conditioned to one type of bedding for 24 h, during which each rat was pair‐housed with a weight‐, age‐, and sex‐matched conspecific in a compartment with an arbitrarily assigned bedding type. On Day 3, experimental rats were moved to an isolated cage (CS−) with the other type of bedding for 24 h to undergo the isolation conditioning. Bedding assignments were counterbalanced for an unbiased design. On Day 4, animals were then tested alone for 30 min in the two‐chambered box to determine post‐conditioning preference for either type of bedding. The time spent in each chamber was recorded as a measure of social conditioned place preference, and a social preference index was calculated by dividing the time spent in the chamber containing the bedding used for the social conditioning (CS+) by the time spent in both chambers (CS+ and CS−) during the 30 min post‐conditioning test. A preference ratio of 0.5 indicated no preference for either compartment, with ratios above 0.5 designating a preference and ratios below 0.5 designating an aversion. Fresh bedding was used at each step, and chambers were thoroughly cleaned between sessions with ethanol 70% to avoid olfactory confounders. The experiments were conducted between 10:00 a.m. and 2:30 p.m.

#### 2.3.2 Three‐Chamber Test

The test was performed at PNDs 35–45 as previously described (Schiavi et al. 2023). The apparatus was a rectangular three‐chamber box with two lateral chambers (30 cm × 35 cm × 35 cm; l × w × h) connected to a central chamber (15 cm × 35 cm × 35 cm; l × w × h). Each lateral chamber contained a small Plexiglass round wire cage (16 cm (diameter), 33 cm (height)) with a bar grid to enclose the stimulus animal and maximize socialization by sniffing between the experimental rat and the stimulus. The stimulus and the experimental rat were not cage mates. Stimulus animals were purchased from an authorized provider (Charles River, Italy) and housed separately from the experimental groups to ensure unfamiliarity and avoid prior social interaction, which could confound sociability in the three‐chamber test. In the habituation phase, each experimental rat was individually allowed to explore a three‐chamber apparatus for 10 min and then confined in the central compartment. An unfamiliar stimulus animal was confined in a cage located in one chamber of the apparatus, while the cage in the other chamber was left empty. Both doors to the side chambers were then opened, allowing the experimental animal to explore the apparatus for 10 min. The time spent in social approach (sniffing the stimulus animal) and the time spent exploring the empty chamber were scored. The discrimination index was calculated as the difference in time spent by each animal sniffing the cage with the stimulus animal compared with the empty cage divided by the total time spent exploring both cages in percentage.

#### 2.3.3 Social Interaction Test

Social interaction was assessed as previously described (Schiavi et al. 2020). The test was performed in a sound‐attenuated chamber under dim light conditions in adult rats (PND 75–85). The testing arena consisted of a Plexiglas cage measuring 45 × 45 cm, with 2 cm of wood shavings covering the floor. Rats were individually habituated to the test cage for 5 min on each of the 2 days prior to testing. Before testing, animals were socially isolated for 24 h to enhance their social motivation and thus facilitate the expression of social behaviors during testing. Behavior was assessed per pair of animals and analyzed by a trained observer who was unaware of treatment conditions. The test consisted of placing two animals from the same experimental group (i.e., both from CTRL or both from ESI) into the test cage for 10 min. The animals in a pair did not differ by more than 10 g in body weight; furthermore, they were housed in different cages and therefore had no previous common social experience. The total time and frequency of play‐related behaviors (pouncing, pinning, and boxing) and social exploration (sniffing any part of the body of the test partner, social grooming, following/chasing, crawling under/over, and kicking) were scored per pair of animals (Bara et al. 2018).

#### 2.3.4 Forced Swimming Test

The forced swimming test was used to evaluate depressive‐like phenotype (Armario 2021). Rats at PNDs 75–85 were forced to swim in a tank (50 cm in height × 20 cm in diameter), filled to a depth of 30 cm with water at a temperature of 25°C. The water depth prevented the animals from reaching the bottom of the container with their hind limbs or tail. The rats were gently placed in the water and allowed to swim for 15 min (pre‐test session). At the end of the 15‐min swimming period, rats were removed from the water and were immediately and gently wiped to dryness with absorbent paper before they were returned to the home cage. The day after, the animals were re‐exposed to the same tank for 5 min (test session). During the test, time spent swimming, floating, and climbing was scored. Floating is defined as the minimum movement needed to keep the animal's head above water.

### 2.4 Tissue Collection

Another cohort of male and female rats exposed to the same isolation protocol described in Section 2.2 was decapitated at PND 35 and PND 75 to perform molecular analyses at adolescence and adulthood, respectively. Brains were quickly removed and placed into an ice‐cold plate. NAc was dissected under stereomicroscope as a coronal section (from Bregma +2.76 to Bregma +0.84 mm), according to the Rat Brain atlas (Paxinos and Watson 2013) and stored at −80°C until analysis.

### 2.5 Western Blot Analysis

Protein samples in the crude membrane fraction were extracted from NAc tissues as previously described (Caffino et al. 2018). The total amount of proteins in the crude membrane fraction was quantified by the Bio‐Rad Protein Assay (Bio‐Rad Laboratories). For each sample, 8 micrograms of proteins were run on a sodium dodecyl sulfate‐14% polyacrylamide gel and then electrophoretically transferred onto a nitrocellulose membrane (Bio‐Rad Laboratories). Blots were cut close to the expected molecular weight of the protein of interest, then blocked with I‐Block solution (Life Technologies Italia) in TBS + 0.1% Tween‐20 buffer for 1 h at room temperature. Each blot was probed overnight at 4°C with antibodies against the phosphorylated forms of proteins, then stripped and incubated with the antibodies specific for the respective total form. The conditions of the primary antibodies were the following: anti‐mBDNF (1:500, RRID: AB_2927780); anti‐pTrkB_Y706_ (1:200, RRID: AB_3339831); anti‐TrkB (1:1000, RRID: AB_2155125); anti‐Rab5 (1:1000, RRID: AB_832625); anti‐Rab11 (1:750, RRID: AB_10693925); anti‐pAKT_S473_ (1:1000, RRID: AB_2315049); anti‐AKT (1:1000, RRID: AB_329827); anti‐pERK2_T185/Y187_ (1:1000, RRID: AB_2315112); anti‐ERK2 (1:2000, RRID: AB_390779); anti‐β − actin (1:10000, RRID: AB_476744). Immunocomplexes were visualized by chemiluminescence by means of the Chemidoc MP Imaging System (Bio‐Rad Laboratories) and analyzed with Image LabTM software (Bio‐Rad), using Optical Density as unit of measurement. Each result was standardized to the β − actin control protein. Representative immunoblots for each protein are shown in Figures 5H, 6D, 7H, 8D, while cropped immunoblots related to the expression levels of the proteins analyzed are shown in Figure S1. As gels were run at least twice, a correction factor was used to average the results from the gels: correction factor gel B = average of (OD protein of interest/OD β‐actin for each sample loaded in gel A)/(OD protein of interest/OD β‐actin for the same sample loaded in gel B) (Caffino, Verheij, et al. 2020).

### 2.6 Data Analysis and Statistics

The data and statistical analyses comply with the recommendations on experimental design and analysis in pharmacology (Curtis et al. 2018). All studies were designed to generate groups of equal size using blinded analysis. The minimum number of animals and sample sizes required to achieve statistical significance were determined by power analysis and prior experience, assuming 80% power at a significance level of 0.05. Due to litter constraints, in the social interaction experiment shown in Figure 3C,D, the ESI male group included *n* = 4 pairs (8 animals). Data are expressed as mean ± SEM. To assess the effects of ESI in male and female rats, data were analyzed by two‐way ANOVA, with sex and isolation conditions as factors. Normality of data was conducted by the Shapiro–Wilk test and the data met the assumption of normality according to the specified statistical test. The paired *t*‐test was used to determine post‐conditioning preference for either type of bedding in the social‐induced CPP task for each experimental group (Table 1). The accepted value for significance was set at *p* < 0.05. The Tukey's post hoc test was used for individual group comparisons. Post hoc tests were only conducted when F in ANOVA achieved *p* < 0.05 and there was no significant inhomogeneity of variance. For both behavioral and molecular experiments, subjects were eliminated from the final dataset when detected as significant outliers by Grubb's test. Data were analyzed using GraphPad Prism Software (Version 10.5.0).

**TABLE 1:** Table 1 shows the time spent by each experimental group (CTRL and ESI female and male rats at adolescence (PND 35–45) and adulthood (PND 75–85)) in the chamber paired with social interaction (CS+) and chamber associated with social isolation (CS–) during post‐conditioning in the socially‐induced conditioned place preference (sCPP) task and their comparison by paired *t*‐test.

Conditioning	Sex	Post‐natal day	Time CS + in sec (Mean ± SEM)	Time CS—in sec (Mean ± SEM)	Paired *t* test	*p*
CTRL	Female	35–45	950.9 ± 35.78	849.1 ± 35.78	*t* = 1.423, df = 9	0.1885
ESI	Female	35–45	973.7 ± 72.05	826.3 ± 72.05	*t* = 1.023, df = 9	0.333
CTRL	Male	35–45	1071 ± 54.09	728.6 ± 54.09	*t* = 3.168, df = 7	0.0157*
ESI	Male	35–45	944.1 ± 108.7	855.9 ± 108.7	*t* = 0.4059, df = 7	0.6969
CTRL	Female	75–85	1035 ± 97.54	765 ± 97.54	*t* = 1.384, df = 5	0.2249
ESI	Female	75–85	969.7 ± 36.13	830.3 ± 36.13	*t* = 1.928, df = 5	0.1118
CTRL	Male	75–85	1244 ± 74.26	555.8 ± 74.26	*t* = 4.636, df = 7	0.0024**
ESI	Male	75–85	446 ± 62.78	1354 ± 62.78	*t* = 7.232, df = 5	0.0008***

*Note:* *: *p*<0.05; **: *p*<0.01; ***: *p*<0.001.

## 3 Results

### 3.1 Behavioral Results

#### 3.1.1 Effect of ESI on Socially‐Induced Conditioned Place Preference (sCPP) in Female and Male Rats Across Development

**FIGURE 2:**
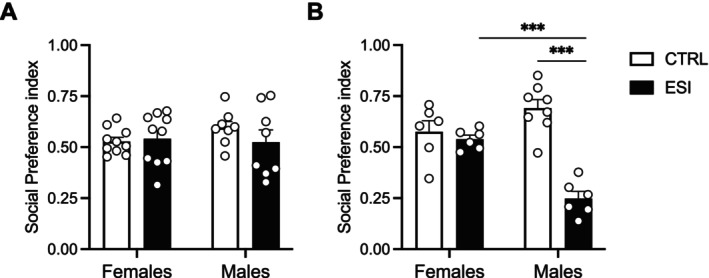
Effect of ESI in socially‐induced Conditioned Place Preference (sCPP) in male and female rats across development. No significant differences were found between male and female CTRL and ESI rats at adolescence in the social preference index (A). At adulthood, ESI male rats showed a significant reduction in the social preference index compared to CTRL male and ESI female rats (B). Data (*n* = 6–10/group/sex) represent mean values ± SEM; ***p < 0.001 (two‐way ANOVA followed by Tukey post hoc test). CTRL, controls; ESI, early social isolation.

To assess whether ESI altered social reward processing in a sex‐dependent manner, we tested independent groups of female and male CTRL and ESI rats at adolescence and adulthood in the sCPP, which relies on the preference of the animal to choose between a contextual cue previously associated with a social stimulus and a distinct set of contextual cues (Trezza et al. 2009; Manduca et al. 2021). At adolescence, two‐way ANOVA for the social preference index (Figure 2A) did not show a significant effect of sex (F_(1,32)_ = 0.417, *p* = 0.523), ESI (F_(1,32)_ = 0.551, *p* = 0.463), and sex x ESI interaction (F_(1,32)_ = 1.137, *p* = 0.294). When time spent in the CS+ and CS– chambers was analyzed to determine post‐conditioning preference (Table 1), we found that only CTRL male rats showed sCPP as they spent more time in the CS+ than CS− chamber (*t* = 3.168, df = 7; *p* < 0.05 paired *t*‐test), without significant differences in the other experimental groups.

At adulthood, two‐way ANOVA revealed a significant effect of sex (F_(1,22)_ = 4.630, *p* < 0.05), ESI (F_(1,22)_ = 34.93, *p* < 0.0001) and sex x ESI interaction (F_(1,22)_ = 25.16, *p* < 0.0001) (Figure 2B), suggesting a detrimental effect of ESI on social reward processing in male rats at adulthood. Post hoc analysis revealed a significant reduction in the social preference index in ESI male rats compared to CTRL male rats (*p* < 0.001) and a significant difference between ESI males and females (*p* < 0.001), highlighting sex differences at adulthood in social reward processing following ESI. When time spent in each chamber was analyzed to determine post‐conditioning preference (Table 1), we found that CTRL male rats spent more time in the CS+ than CS− chamber (*t* = 4.636, df = 7; *p* < 0.01), whereas ESI male rats showed social aversion since they spent more time in the CS− than CS+ chamber (*t* = 7.232, df = 5; *p* < 0.001). No significant differences were found in female rats.

#### 3.1.2 Effect of ESI in the Three‐Chamber Test at Adolescence and Social Interaction at Adulthood in Female and Male Rats

**FIGURE 3:**
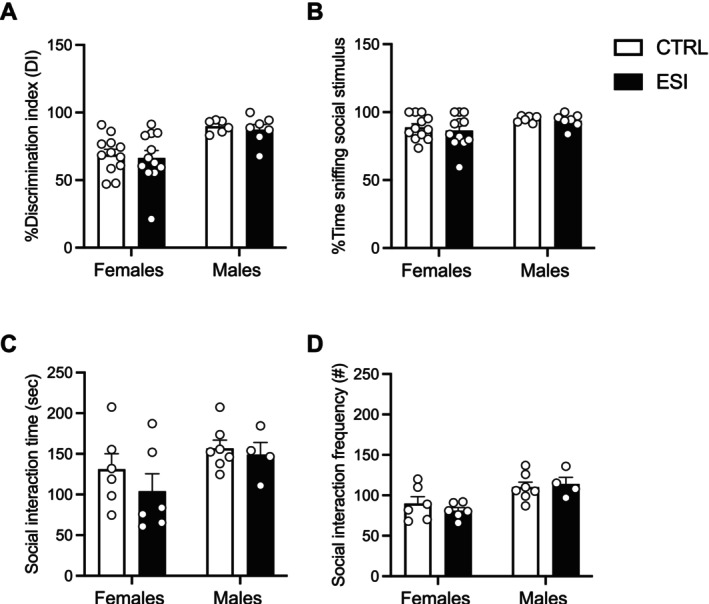
Effect of ESI in the three‐chamber test and social interaction in male and female rats across development. No significant differences were found between CTRL and ESI rats at adolescence in the % discrimination index (A) and the % time spent sniffing the social stimulus (B) in the three‐chamber test. Regardless of the experimental condition (CTRL and ESI), male and female rats showed no differences in the total time (C) and frequency (D) of dyadic social interaction at adulthood. Data (*n* = 4–12/group/sex) represent mean values ± SEM (two‐way ANOVA followed by Tukey post hoc test). CTRL, controls; ESI, early social isolation.

To evaluate whether the deficits in social reward processing observed in the sCPP task extended to other components of the social repertoire, we first tested animals in the three‐chamber test at adolescence to evaluate their sociability. Two‐way ANOVA for the discrimination index in percentage (Figure 3A) showed a significant effect of sex (F_(1,33)_ = 17.92, *p* = 0.0002), but not ESI (F_(1,33)_ = 0.245, *p* = n.s.) and sex x ESI interaction (F_(1,33)_ = 0.0001, *p* = n.s.). Similarly, two‐way ANOVA for the percentage of time sniffing the social stimulus (Figure 3B) showed a significant effect of sex (F_(1,33)_ = 4.631, *p* = 0.0388), but not a main effect of ESI (F_(1,33)_ = 0.358, *p* = n.s.) and sex x ESI interaction (F_(1,33)_ = 0.038, *p* = n.s.). Overall, these results showed no significant effect of ESI on sociability in the three‐chamber test in both male and female rats at adolescence.

When tested at adulthood, no difference was found in the total time (Figure 3C) and frequency (Figure 3D) of social interaction during a dyadic free social interaction session. Two‐way ANOVA for the time spent in social interaction showed no main effect of sex (F_(1,19)_ = 4.106, *p* = n.s.), ESI (F_(1,19)_ = 1.005, *p* = n.s.) and sex x ESI interaction (F_(1,19)_ = 0.302, *p* = n.s.) (Figure 3C). Moreover, two‐way ANOVA for the number of social events showed a main effect of sex (F_(1,19)_ = 14.20, *p* = 0.0013), but not ESI (F_(1,19)_ = 0.117, *p* = n.s.) and sex x ESI interaction (F_(1,19)_ = 0.827, *p* = n.s.) (Figure 3D). Interestingly, these results are in line with our recent findings showing that ESI male and female rats had no deficits in social play behavior at adolescence (Mottarlini, Rizzi, et al. 2024), suggesting that dyadic social interaction is preserved across development in the ESI animals.

#### 3.1.3 Effect of ESI on Depressive‐Like Behavior in Adult Female and Male Rats

**FIGURE 4:**
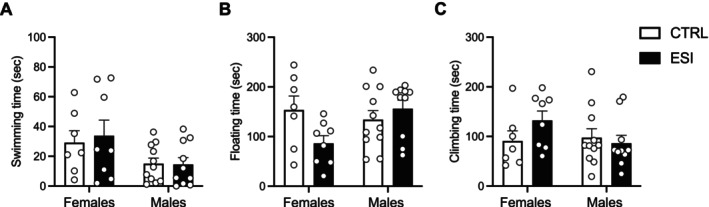
Effect of ESI on depressive‐like behavior in male and female rats in adulthood. No significant effects of social isolation in early life were found in the time spent swimming (A), floating (B) and climbing (C) in the Forced Swimming Test in male and female rats in adulthood. Data (*n* = 7–11/group/sex) represent mean values ± SEM (two‐way ANOVA followed by Tukey post hoc test). CTRL, controls; ESI, early social isolation.

Exposure to early life stress is a significant risk factor for mental illnesses such as major depressive disorder (Ochi and Dwivedi 2023). To evaluate whether our ESI protocol induced depressive‐like behavior in adulthood in a sex‐specific manner, we used the forced swimming test, a widely used model for assessing a depressive‐like phenotype in rodents. Our results showed that adult ESI female and male rats did not display depressive‐like behaviors when compared to CTRL animals (Figure 4A–C). Two‐way ANOVA for the time spent swimming (Figure 4A) showed a significant effect of sex (F_(1,32)_ = 6.343, *p* = 0.0170), but no significant effect of ESI (F_(1,32)_ = 0.092, *p* = n.s.) and sex x ESI interaction (F_(1,32)_ = 0.145, *p* = n.s.). Two‐way ANOVA for the time floating (Figure 4B) revealed no effect of sex (F_(1,32)_ = 1.617, *p* = n.s.), ESI (F_(1,32)_ = 1.305, *p* = n.s.) and a main effect of sex x ESI interaction (F_(1,32)_ = 5.087, *p* = 0.0311). Post hoc analysis revealed a trend (*p* = 0.076) between female and male ESI rats in the time spent floating. Moreover, two‐way ANOVA for the time spent climbing (Figure 4C) revealed no significant effect of sex (F_(1,32)_ = 1.122, *p* = n.s.), ESI (F_(1,32)_ = 0.673, *p* = n.s.) and sex x ESI interaction (F_(1,32)_ = 2.039, *p* = n.s.). Overall, these results suggested that brief periods of repeated social isolation during the third postnatal week did not induce depressive‐like behavior in adulthood in female and male rats.

### 3.2 Molecular Results

#### 3.2.1 Effect of ESI on BDNF Signaling and Endocytic Mechanisms in the NAc of Adolescent Animals

**FIGURE 5:**
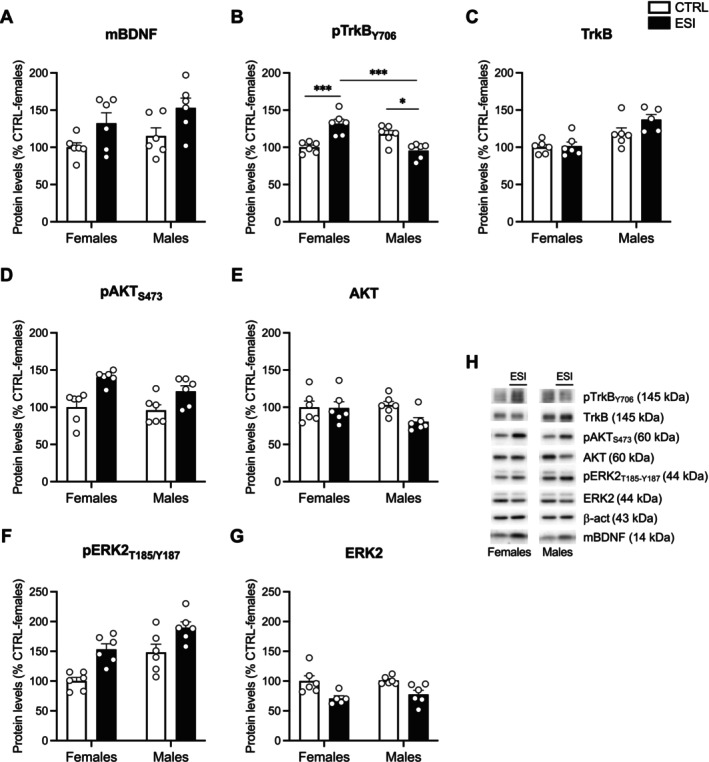
Effect of ESI on mBDNF and its downstream signaling in the nucleus accumbens of adolescent rats. Protein levels of mBDNF (A), pTrkB_Y706_ (B), TrkB (C), pAKT_S473_ (D), AKT (E), pERK2_T185/Y187_ (F) and ERK2 (G) measured in the crude membrane fraction of NAc at PND 35 in female and male rats that underwent ESI (PND 14‐PND 21). Representative immunoblots for each protein and β‐Actin are shown in panel (H). Data (*n* = 5–6/group/sex) are expressed in scatter plot bar graphs as percentages of female CTRL and represent the mean ± SEM; *p < 0.05, ***p < 0.001 (two‐way ANOVA followed by Tukey post hoc test). CTRL, controls; ESI, early social isolation; NAc, nucleus accumbens; PND, post‐natal day.

To assess whether ESI was associated with a dysregulation of neuroplastic mechanisms at adolescence, we first investigated the BDNF pathway in the crude membrane fraction of the NAc in both sexes at PND 35. Two‐way ANOVA of mBDNF protein levels showed a significant effect of ESI (F_(1,20)_ = 9.163, *p* = 0.0067), but not of sex (F_(1,20)_ = 2.386, *p* = n.s.) and sex x ESI interaction (F_(1,20)_ = 0.05146, *p* = n.s.) (Figure 5A). The analysis of pTrkB_Y706_, the active form of the high affinity BDNF receptor, revealed no effect of sex (F_(1,20)_ = 3.864, *p* = n.s.) and ESI (F_(1,20)_ = 1.061, *p* = n.s.) but a main effect of their interaction (F_(1,20)_ = 32.39, *p* < 0.0001) (Figure 5B). Post hoc analyses highlighted an opposite response to the ESI condition depending on the sex: in fact, pTrkB_Y706_ protein levels were increased in females while reduced in males compared to their respective CTRLs (Figure 5B). Two‐way ANOVA of the total form of the TrkB protein levels showed a significant effect of sex (F_(1,19)_ = 19.81, *p* = 0.0003) but not of ESI (F_(1,19)_ = 2.831, *p* = n.s.) or sex x ESI interaction (F_(1,19)_ = 1.982, *p* = n.s.) (Figure 5C).

Since activation of the BDNF/TrkB pathway induces downstream effectors, such as AKT and ERK2, we evaluated whether the opposite activation of TrkB in females and males following the ESI procedure might lead to a different activation of these two kinases. Two‐way ANOVA of pAKT_S437_ revealed a significant effect of ESI (F_(1,20)_ = 23.44, *p* < 0.0001), while no effect of sex (F_(1,20)_ = 1.341, *p* = n.s.) and of sex x ESI interaction (F_(1,20)_ = 2.522, *p* = n.s.) (Figure 5D). No main effect of AKT total protein levels was revealed by two‐way ANOVA analysis (sex: F_(1,20)_ = 1.211, *p* = n.s.; ESI: F_(1,20)_ = 2.801, *p* = n.s.; sex x ESI: F_(1,20)_ = 2.219, *p* = n.s.) (Figure 5E). Concerning pERK2_T185/Y187_ protein levels, two‐way ANOVA showed main effects of sex (F_(1,20)_ = 17.13, *p* = 0.0005) and ESI (F_(1,20)_ = 21.45, *p* = 0.0002) per se but not of their interaction (F_(1,20)_ = 0.3192, *p* = n.s.) (Figure 5F). Two‐way ANOVA of ERK2 protein levels revealed a significant effect of ESI (F_(1,19)_ = 17.15, *p* = 0.0006), but not of sex (F_(1,19)_ = 0.3488, *p* = n.s.) and sex x ESI interaction (F_(1,19)_ = 0.1948, *p* = n.s.) (Figure 5G).

**FIGURE 6:**
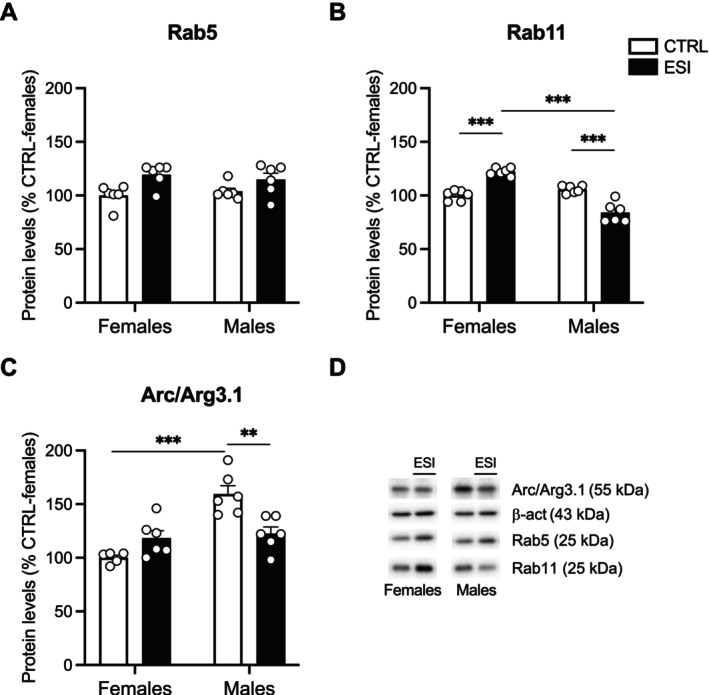
Effect of ESI on Rab‐mediated endocytic pathway in the nucleus accumbens of adolescent rats. Protein levels of Rab5 (A), Rab11 (B) and Arc/Arg3.1 (C) measured in the crude membrane fraction of NAc at PND 35 in female and male rats that underwent ESI (PND 14‐PND 21). Representative immunoblots for each protein and β‐Actin are shown in panel (D). Data (*n* = 5–6/group/sex) are expressed in scatter plot bar graphs as percentages of female CTRL and represent the mean ± SEM; **p < 0.01, ***p < 0.001 (two‐way ANOVA followed by Tukey post hoc test). CTRL, controls; ESI, early social isolation; NAc, nucleus accumbens; PND, post‐natal day.

To modulate neuroplastic mechanisms, BDNF/TrkB signaling interacts with Rab‐mediated endocytic pathways (Moya‐Alvarado et al. 2022). Rab5 internalizes receptors from the membrane to early endosomes, while Rab11 recycles them back into the membrane (Langemeyer et al. 2018). Two‐way ANOVA of Rab5 protein levels showed a significant effect of ESI (F_(1,20)_ = 11.72, *p* = 0.0027), while there were no effects of either sex (F_(1,20)_ = 0.005786, *p* = n.s.) and sex x ESI interaction (F_(1,20)_ = 0.8856, *p* = n.s.) (Figure 6A). Two‐way ANOVA of Rab11 protein levels revealed no effects of ESI (F_(1,20)_ = 0.02144, *p* = n.s.), while there were significant effects of sex (F_(1,20)_ = 48.69, *p* < 0.0001) and of sex x ESI interaction (F_(1,20)_ = 86.69, *p* < 0.0001) (Figure 6B). Post hoc analyses unraveled an opposite response to the ESI condition in female and male animals: Rab11 was increased in females while reduced in males compared to their respective CTRLs (Figure 6B).

Next step was to investigate whether the observed alterations could be due, at least in part, to changes in the stabilization of synaptic functions. Thus, we measured the expression of the activity‐regulated cytoskeletal‐associated protein, Arc/Arg3.1. Two‐way ANOVA of Arc/Arg3.1 protein levels revealed a main effect of sex (F_(1,19)_ = 23.61, *p* = 0.0001) and a significant effect of their interaction (F_(1,19)_ = 17.68, *p* = 0.0005), but no effect of ESI (F_(1,19)_ = 2.012, *p* = n.s.) (Figure 6C). Interestingly, male CTRL rats showed enhanced basal Arc/Arg 3.1 protein levels in respect to female CTRL animals. Moreover, female adolescent rats did not change Arc/Arg 3.1 protein levels in response to ESI, whereas males exposed to ESI significantly decreased their levels compared to male CTRL rats (Figure 6C).

#### 3.2.2 Effect of ESI on BDNF Signaling and Endocytic Mechanisms in the NAc of Adult Animals

To investigate whether the exposure to early life stress could have long‐lasting molecular scars, we performed the same analyses described in the previous paragraph (3.2.1) in the NAc of female and male rats at PND 75.

**FIGURE 7:**
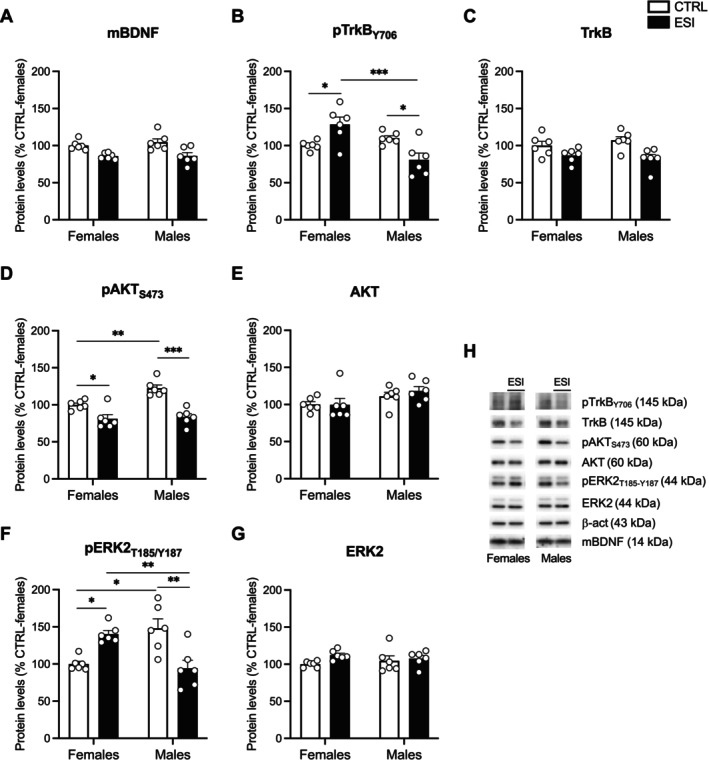
Effect of ESI on mBDNF and its downstream signaling in the nucleus accumbens of adult rats. Protein levels of mBDNF (A), pTrkB_Y706_ (B), TrkB (C), pAKT_S473_ (D), AKT (E), pERK2_T185/Y187_ (F) and ERK2 (G) measured in the crude membrane fraction of NAc at PND 75 in female and male rats that underwent ESI (PND 14‐PND 21). Representative immunoblots for each protein and β‐Actin are shown in panel (H). Data (*n* = 6/group/sex) are expressed in scatter plot bar graphs as percentages of female CTRL and represent the mean ± SEM; *p < 0.05, **p < 0.01, ***p < 0.001 (two‐way ANOVA followed by Tukey post hoc test). CTRL, controls; ESI, early social isolation; NAc, nucleus accumbens; PND, post‐natal day.

Two‐way ANOVA of mBDNF revealed a significant effect of ESI (F_(1,20)_ = 19.99, *p* = 0.0002), but not of sex (F_(1,20)_ = 0.4003, *p* = n.s.) or sex x ESI interaction (F_(1,20)_ = 0.3353, *p* = n.s.) (Figure 7A). Two‐way ANOVA of pTrkB_Y706_ levels showed no effects of ESI (F_(1,20)_ = 0.001092, *p* = n.s.), while significant effects of sex (F_(1,20)_ = 7.240, *p* = 0.0141) and sex x ESI (F_(1,20)_ = 16.93, *p* = 0.0005) (Figure 7B). Similarly to adolescent animals, post hoc testing unraveled an opposite response to ESI condition between female and male animals at PND 75: ESI increased pTrkB_Y706_ protein levels in females, while it reduced its levels in males (Figure 7B). Two‐way ANOVA of total TrkB protein levels highlighted only a significant effect of ESI (F_(1,20)_ = 13.71, *p* = 0.0014), while no effects of sex (F_(1,20)_ = 0.07117, *p* = n.s.) and of sex x ESI were observed (F_(1,20)_ = 1.259, *p* = n.s.) (Figure 7C).

Then, we analyzed the BDNF effectors AKT and ERK2 in adult male and female rats. Two‐way ANOVA of pAKT_S473_ revealed significant effects of ESI (F_(1,20)_ = 43.17, *p* < 0.0001), sex (F_(1,20)_ = 8.064, *p* = 0.0101), and sex x ESI interaction (F_(1,20)_ = 4.855, *p* = 0.0395) (Figure 7D). Male CTRL animals displayed basal enhanced pAKT_S473_ versus female CTRL rats. Exposure to ESI reduced pAKT_S473_ protein levels in both female and male rats compared to their respective CTRLs (Figure 7D). Conversely to pAKT_S473_, two‐way ANOVA of AKT showed a main effect of sex (F_(1,20)_ = 5.469, *p* = 0.0299) but not of ESI (F_(1,20)_ = 0.2787, *p* = n.s.) and sex x ESI (F_(1,20)_ = 0.3695, *p* = n.s.) (Figure 7E). Two‐way ANOVA of pERK2_T185/Y187_ levels showed no effects of sex (F_(1,20)_ = 0.8338, *p* = n.s.) and ESI (F_(1,20)_ = 0.001296, *p* = n.s.) per se, whereas a significant effect of their interaction (F_(1,20)_ = 25.55, *p* < 0.0001) (Figure 7F). As observed for pAKT_S473_ in male CTRL rats, pERK2_T185/Y187_ levels were increased; ESI increased pERK2_T185/Y187_ levels in females, whereas males showed reduced levels compared to both female ESI and male CTRL rats (Figure 7F). Two‐way ANOVA of ERK2 did not report any effect of sex (F_(1,20)_ = 0.0002087, *p* = n.s.), ESI (F_(1,20)_ = 3.074, *p* = n.s.) or sex x ESI interaction (F_(1,20)_ = 1.124, *p* = n.s.) (Figure 7G).

**FIGURE 8:**
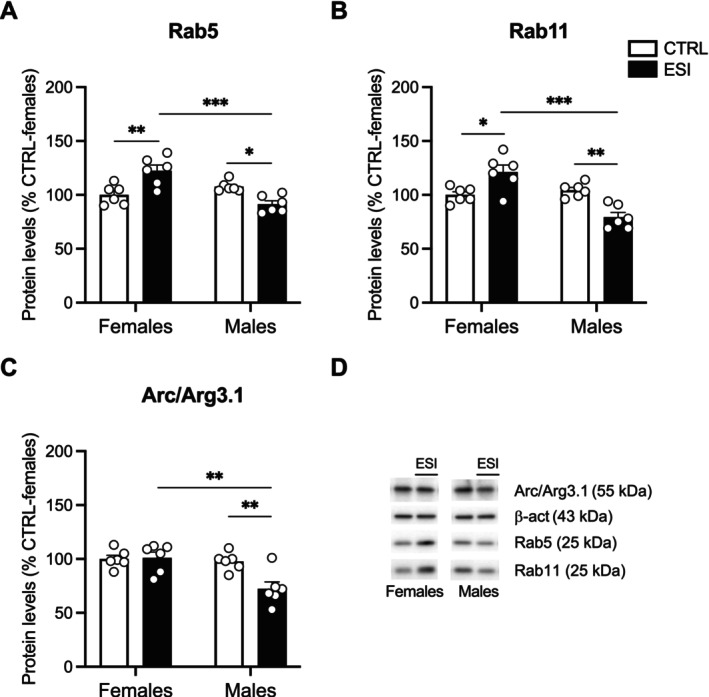
Effect of ESI on Rab‐mediated endocytic pathway in the nucleus accumbens of adult rats. Protein levels of Rab5 (A), Rab11 (B) and Arc/Arg3.1 (C) measured in the crude membrane fraction of NAc at PND 75 in female and male rats that underwent ESI (PND 14‐PND 21). Representative immunoblots for each protein and β‐Actin are shown in panel (D). Data (*n* = 6/group/sex) are expressed in scatter plot bar graphs as percentages of female CTRL and represent the mean ± SEM; *p < 0.05, **p < 0.01, ***p < 0.001 (two‐way ANOVA followed by Tukey post hoc test). CTRL, controls; ESI, early social isolation; NAc, nucleus accumbens; PND, post‐natal day.

Last, Rab‐mediated endocytic‐recycling mechanisms were also analyzed. Two‐way ANOVA of Rab5 unraveled no effect of ESI (F_(1,20)_ = 0.5624, *p* = n.s.), but significant effects of sex (F_(1,20)_ = 9.235, *p* = 0.0065) and sex x ESI interaction (F_(1,20)_ = 25.87, *p* < 0.0001) (Figure 8A). In line with pTrkB_Y706_ levels, post hoc testing showed an increased expression of Rab5 in female rats exposed to ESI, whereas males showed reduced levels compared to both their respective CTRL and female ESI group (Figure 8A). Two‐way ANOVA of Rab11 showed no effect of ESI (F_(1,20)_ = 0.1773, *p* = n.s.), but significant effects of sex (F_(1,20)_ = 17.84, *p* = 0.0004) and sex x ESI interaction (F_(1,20)_ = 27.01, *p* < 0.0001) (Figure 8B). Similarly to Rab5, post hoc analyses of Rab11 showed that females subjected to ESI displayed increased Rab11 protein levels compared to female CTRL rats; conversely, males that underwent ESI showed reduced protein levels in relation to both male CTRL and female ESI rats (Figure 8B). Two‐way ANOVA of Arc.Arg3.1 revealed significant effects of ESI (F_(1,20)_ = 6.246, *p* = 0.0213), sex (F_(1,20)_ = 10.53, *p* = 0.0041) and their interaction (F_(1,20)_ = 7.432, *p* = 0.0130) (Figure 8C). Post hoc testing showed no differences among female CTRL and ESI. Conversely, ESI male animals displayed reduced Arc.Arg3.1 protein levels compared to both male CTRL and female ESI (Figure 8C).

## 4 Discussion

The present study demonstrated that exposure to brief social isolation periods during the third post‐natal week in rats is associated with sex‐specific enduring behavioral and molecular changes. Specifically, we found that ESI induced deficits in social reward processing in male rats, together with sex‐dependent effects on the BDNF/TrkB signaling pathway and alterations of vesicular sorting in the NAc.

Recent preclinical research provided valuable insights into the effects of social development under isolated environments (Xiong et al. 2023); however, the sex‐specific impact of ESI on social behavior and neuroplastic mechanisms in the brain reward system remains poorly understood. One of the most common experimental approaches to study the neurobiological underpinnings of social reward in rodents is the conditioned place preference (CPP) paradigm. This experimental approach, based on classical conditioning, measures the rewarding properties of social interaction and social reward learning (Trezza et al. 2011; Panksepp and Lahvis 2007; Manduca et al. 2021). To assess whether ESI altered social reward processing in a sex‐dependent manner, we tested female and male CTRL and ESI‐exposed rats at adolescence and adulthood in the sCPP task. At adolescence, we did not find a significant difference in the social preference index between groups; however, only CTRL male rats showed sCPP since they spent more time in the social conditioning bedding, confirming that social interactions are rewarding in adolescent male rats (Panksepp and Lahvis 2007; Dolen et al. 2013; Gunaydin et al. 2014). At adulthood, ESI‐exposed male rats showed a significant reduction in the social preference index compared to CTRL male rats, indicating social aversion since they preferred the isolation conditioning bedding. Moreover, ESI male rats exhibited a reduced social preference index compared to ESI females, highlighting sex differences at adulthood in social reward processing following ESI. Together, our findings suggested that ESI altered the sensitivity to the positive subjective and reinforcing properties of social reward with pronounced effects in adult male rats. Unexpectedly, we failed to detect sCPP in female rats at adolescence and adulthood. Following described procedures (Wei et al. 2015; Dolen et al. 2013; Schiavi et al. 2019), we opted for a 30‐min testing to allow sufficient time for the animals to explore both CS+ and CS– chambers and express a post‐conditioning preference for either type of bedding. This duration balances the need for a reliable measurement of motivational behavior while minimizing potential confounds such as fatigue, habituation, or potential effects of novelty. Protocols with short post‐conditioning sessions are also used, with some methodological differences from our protocol. For instance, Weiss and colleagues used a 10‐day sCPP protocol (i.e., rats were conditioned from Days 2 to 9 and at Day 10 tested in a 15‐min post‐conditioning session), showing that neither individually‐ nor pair‐housed adolescent females showed social CPP (Weiss et al. 2015). The same protocol induced sCPP in male adolescent rats when individually housed, but not when pair‐housed (Yates et al. 2013). Moreover, Douglas and colleagues conditioned the animals for 5 days, with the test session being conducted 24 h after the last conditioning trial, and they showed that isolated animals of both sexes and ages (i.e., adolescence and adulthood) developed sCPP, with the strongest preference emerging in adolescent males (Douglas et al. 2004). In the present work, we used a protocol of 4 days of conditioning, and we acknowledge that even though social contact is rewarding, the effect might be dependent on the context, including the number of conditioning sessions, the preference for contextual cues, and time of post‐conditioning. Moreover, investigating whether changes in the incentive value of social interaction during conditioning could modify the acquisition of sCPP in female rats deserves further investigation. Finally, it would be incremental to test whether the observed deficits in sCPP in male rats were specific to social reward or if they extended to other types of reward, such as drug‐ or food‐induced CPP.

Conversely, general social behavior was not significantly altered by ESI exposure. For instance, in the three‐chamber test at adolescence, there was no effect of ESI on the ability to discriminate between a conspecific and an empty cage, indicating that sociability was preserved across sex and experimental conditions. Similarly, no significant effects of ESI on dyadic social interaction were observed at adulthood, which further supports the idea that ESI specifically alters the rewarding value of social interactions rather than the capacity to engage in social behaviors. This evidence is in line with our recent work (Mottarlini, Rizzi, et al. 2024) showing no deficits in social play behavior at adolescence in male and female rats exposed to this ESI protocol, suggesting that ESI‐induced deficits in social reward processing did not extend to a general impairment in social approach behavior.

Social isolation is a well‐established model for inducing depressive‐like behavior in rodents (Grigoryan et al. 2022). However, our study did not reveal significant changes in the forced swimming test, indicating that ESI did not lead to depressive‐like behaviors in adult male or female rats. This lack of effect may be attributed to the specific ESI protocol used (i.e., 30 min daily from PNDs 14–21), which may not have been sufficient to induce a depressive phenotype at adulthood. Depressive‐like behaviors often require prolonged or more intense stress exposure, such as extended maternal separation or chronic social stress. For instance, there is evidence that social isolation lasting up to 3 weeks does not cause behavioral abnormalities in male rats, suggesting that a longer isolation period may be necessary for this paradigm to reliably induce depressive‐like behavior (Gorlova et al. 2018). Previous work has shown that a short period of isolation during adolescence can induce depressive‐like behaviors when assessed immediately after the isolation period (Leussis and Andersen 2008). Therefore, we cannot exclude that performing the forced swimming test in adolescent rats could result in different effects between CTRL and ESI male and female rats, in line with sCPP results.

From a molecular perspective, one of the most compelling findings of our study is the sex‐dependent activation of the BDNF/TrkB signaling pathway in response to ESI. This, together with dysregulated endosomal sorting, may partly explain the observed alterations in social reward processing. BDNF is a key regulator of neuroplasticity, orchestrating adaptive responses to stress in a brain region‐dependent manner (Han et al. 2025). In the NAc, activation of BDNF/TrkB signaling is strictly related to motivated behaviors (Li and Wolf 2015; Caffino et al. 2022) and has been implicated in depressive phenotypes (De Vry et al. 2016; Berton et al. 2006). In our experimental condition, adolescent male rats exposed to ESI showed increased mBDNF levels, yet this increase was not accompanied by enhanced TrkB phosphorylation at Tyr706. Instead, TrkB phosphorylation was reduced, potentially disrupting downstream signaling. Interestingly, intra‐NAc BDNF infusion and elevated BDNF levels following chronic social defeat stress have been shown to increase stress susceptibility and induce social avoidance (Krishnan et al. 2007). Thus, the ESI‐induced increase in mBDNF, combined with reduced TrkB phosphorylation, might contribute to altered social reward processing during adolescence. In adulthood, the entire BDNF/TrkB signaling pathway was downregulated in male rats exposed to ESI. Even though the implications of reverting such alterations still need to be addressed, this effect leads us to hypothesize that ESI in males might reduce the protective role of BDNF in neuronal functionality and survival, thereby facilitating the development of social aversion. In females exposed to ESI, the increased expression of mBDNF was accompanied by enhanced activation of its signaling pathway, including pTrkB, pAKT, and pERK2. Given that TrkB overexpression in the NAc has been linked to enhanced motivation in the forced swim test and antidepressant‐like effects (De Vry et al. 2016), the ESI‐induced increase in TrkB phosphorylation may counteract the negative effects associated with elevated mBDNF, thereby preserving social reward processing in females. Despite the ESI procedure being a brief manipulation early in life, it is able to modify the maturational trajectory of the neuroplastic landscape in the NAc, reducing the trophic support and thereby contributing to deficits in social reward in male, but not female, rats. This striking sex difference in response to ESI suggests that female and male rats may rely on different mechanisms to modulate reward processing following early‐life stress. Although our study does not establish causality, these findings may help explain sex‐dependent differences in social reward processing across development. Moreover, they align with previous studies indicating that sex differences in BDNF/TrkB signaling could contribute to differential vulnerability to stress‐related psychiatric disorders, such as depression (McEwen 2003).

Another novel aspect of our study is the finding that changes in BDNF signaling induced by ESI were accompanied by alterations in endocytic‐recycling mechanisms mediated by Rab GTPases, which are critical for receptor trafficking and synaptic plasticity (Stenmark 2009; Targa et al. 2023). It has been previously demonstrated that the activity of BDNF/TrkB and the endocytic machinery interact; in particular, Rab5‐positive and Rab11‐positive endosomes colocalize with TrkB, contribute to the BDNF‐induced rearrangement of synaptic structure and function (Lazo et al. 2013; Moya‐Alvarado et al. 2018) and play a role in the sustained activation of BDNF intracellular signaling (Gonzalez‐Gutierrez et al. 2020). In our experimental condition, we found that Rab5 and Rab11, two proteins involved in the internalization and recycling of receptors, respectively, were significantly altered in the NAc by ESI in a sex‐dependent manner. In male rats, Rab5 was upregulated while Rab11 was reduced, suggesting that ESI disrupted the endocytic machinery by enhancing TrkB internalization while impairing its recycling both at adolescence and adulthood. This persistent shift may favor TrkB sorting and degradation (Moya‐Alvarado et al. 2022), thus dampening its activity and potentially driving social reward deficits. Conversely, in females, Rab5 and Rab11 were increased in the NAc of both adolescent and adult rats exposed to ESI, potentially promoting the sorting of TrkB via the recycling pathway and thus sustaining its activity (Song et al. 2015). These sex‐specific alterations in the endocytic machinery might underlie the different social‐induced CPP observed in male and female rats exposed to ESI. Notably, since it has been reported that the activation of the Rab5 Rab11‐mediated recycling pathway prolongs BDNF/TrkB signaling activity (Lazo et al. 2013), the ESI‐induced upregulation of Rab5 and Rab11, paralleled by the increased BDNF signaling specifically observed in females, prompts us to hypothesize that this molecular rearrangement might prevent alterations in social reward in females but not in males.

Last, to have a readout of accumbal neuronal activation (Mottarlini, Caffino, et al. 2024) and since downregulation of Rab11 reduces the expression of BDNF‐dependent genes involved in brain plasticity (Gonzalez‐Gutierrez et al. 2020), we evaluated the activity‐regulated cytoskeletal‐associated protein (Arc/Arg3.1) expression, a marker of synaptic plasticity. Interestingly, Arc/Arg3.1 levels were reduced in male rats exposed to ESI at both adolescence and adulthood but remained unchanged in females across development. This decrease, in parallel with BDNF/TrkB downregulation and Rab11 reduction, suggests an overall decline in accumbal neuroplasticity in males following ESI, further pointing to a sex‐specific involvement of the BDNF/TrkB/Rab machinery in response to early‐life stress. In particular, this suggests that ESI may impair neuronal plasticity in males more profoundly than in females, potentially contributing to long‐lasting deficits in reward processing. These findings are in line with previous studies showing sex‐dependent changes in the regulation of neuroplasticity following early‐life stress (Talani et al. 2025).

Overall, our results demonstrate that ESI can induce sex‐dependent alterations in social reward processing and neuroplastic changes that are detectable several weeks after the isolation procedure, with male rats showing pronounced social reward deficits in adulthood and a maladaptive rearrangement of the BDNF/TrkB/Rab pathways in the NAc. While ESI did not significantly alter general social behavior (including sociability at adolescence and dyadic social interaction at adulthood) or induce depressive‐like behavior in adulthood, it selectively impaired social reward processing in adult males.

This study presents some limitations. The isolation paradigms are highly heterogeneous across laboratories, with differences mainly depending on the length of separation, ranging from brief periods of single or repeated separations to complete separation with artificial rearing from birth to weaning (Stupart et al. 2023). Our findings are specific to the ESI protocol used, and different paradigms may yield different outcomes. Additionally, factors such as sex, single vs. litter‐wide isolation, and testing age contribute to variability across studies. While our molecular analyses focused on the NAc, other brain regions involved in social reward processing and stress response, such as the prefrontal cortex, hippocampus, and amygdala, may also contribute to the observed effects and warrant further investigation. Moreover, we are also aware that our experimental approach lacks causal manipulation; however, our findings are the first to provide new insights into how a mild social isolation stress early in life may shape social reward processing, highlighting the importance of sex differences when studying the neurobiological effects of early stress exposure.

Exploring how ESI selectively produces such effects and modulates the response to adverse situations during a sensitive window of brain development could guide future investigation into sex‐dependent mechanisms underlying the response to adverse environmental situations. Indeed, the ESI model in rodents holds significant translational value for clinical applications, allowing us to study how social support systems influence mental health outcomes, particularly regarding neuropsychiatric conditions where social isolation plays a crucial role. Since dysfunction in the BDNF/TrkB signaling and in the endosomal machinery has been involved in several neurodegenerative diseases (Zhang et al. 2021), an in‐depth understanding of their interaction underlying neuronal plasticity will pave the way for the identification of new pharmacological targets able to preserve brain plasticity.

## Author Contributions

**M. Di Trapano:** data curation, investigation, methodology, software, writing – original draft, formal analysis. **V. Buzzelli:** methodology, software, data curation, investigation, writing – original draft, formal analysis. **B. Rizzi:** methodology, software, data curation, investigation, writing – original draft, formal analysis. **F. Mottarlini:** investigation, methodology, software, data curation, writing – original draft. **S. Schiavi:** methodology, software, data curation, investigation. **R. Ciccocioppo:** resources, writing – review and editing, methodology. **L. Fattore:** methodology, resources, writing – review and editing. **P. Romualdi:** methodology, writing – review and editing, resources. **F. Fumagalli:** conceptualization, funding acquisition, project administration, supervision, writing – review and editing. **V. Trezza:** conceptualization, funding acquisition, writing – review and editing, project administration, supervision. **L. Caffino:** conceptualization, funding acquisition, project administration, supervision, writing – original draft, writing – review and editing, data curation, validation, visualization, formal analysis. **A. Manduca:** conceptualization, writing – review and editing, writing – original draft, funding acquisition, project administration, supervision, data curation, validation, visualization, formal analysis.

## Consent

All authors agreed on the authorship and publication of this article.

## Conflicts of Interest

Viviana Trezza is currently the Reviews Handling Editor of the *Journal of Neurochemistry*. All the other authors declare no conflicts of interest.

## Peer Review

The peer review history for this article is available at https://www.webofscience.com/api/gateway/wos/peer‐review/10.1111/jnc.70181.

## Supporting information


**Figure S1:** jnc70181‐sup‐0001‐FigureS1.pdf.

## Data Availability

Data available on request from the authors.
