# Supplementary material for: Early Life Social Isolation Dysregulates Social Reward Processing, BDNF Signaling, and Intracellular Vesicular Sorting in the Nucleus Accumbens of Male and Female Rats
Source: J Neurochem. 2025 Aug 5;169(8):e70181. doi: 10.1111/jnc.70181 (PMC12323299; doi:10.1111/jnc.70181)

Early life social isolation dysregulates social reward processing, BDNF signaling and intracellular vesicular sorting in the nucleus accumbens of male and female rats

Di Trapano M.[1,2], Buzzelli V.[1*], Rizzi B[2,3*], Mottarlini F.[3], Schiavi S.[1], Ciccocioppo R.[4], Fattore L.[5], Romualdi P.[6], Fumagalli F.[3], Trezza V.[1,7], Caffino L.[3*#], Manduca A.[1,7,8*#]

[1]Department of Science, Section of Biomedical Sciences and Technologies, Roma Tre University, Rome, Italy;

[2]Center for Neuroscience, University of Camerino, Camerino, Italy;

[3]Department of Pharmacological and Biomolecular Sciences 'Rodolfo Paoletti', University of Milan, Milan, Italy;

[4]School of Pharmacy, Center for Neuroscience, Pharmacology Unit, University of Camerino, Camerino, Italy;

[5]Research National Council (CNR) Institute of Neuroscience-Cagliari, National Research Council, Cagliari, Italy;

[6]Department of Pharmacy and Biotechnology, University of Bologna, Bologna, Italy;

[7]Neuroendocrinology, Metabolism and Neuropharmacology Unit, IRCCS Fondazione Santa Lucia, Rome, Italy;

[8]Department of Physiology and Pharmacology, Sapienza University of Rome, Rome, Italy.

*: these authors equally contributed.

#: Corresponding authors

Supplementary Figure 1. Cropped immunoblots of pTrkB$_{Y706}$, TrkB, pAKT$_{S473}$, AKT, Arc/Arg3.1, pERK2$_{T185/Y187}$, ERK2, β-act, Rab5, Rab11 and mBDNF measured in the crude membrane fraction of the Nucleus Accumbens of PND 35 and PND 75 female and male rats that underwent early social isolation (ESI) (PND 14-PND 21) and presented in Figures 5-8.

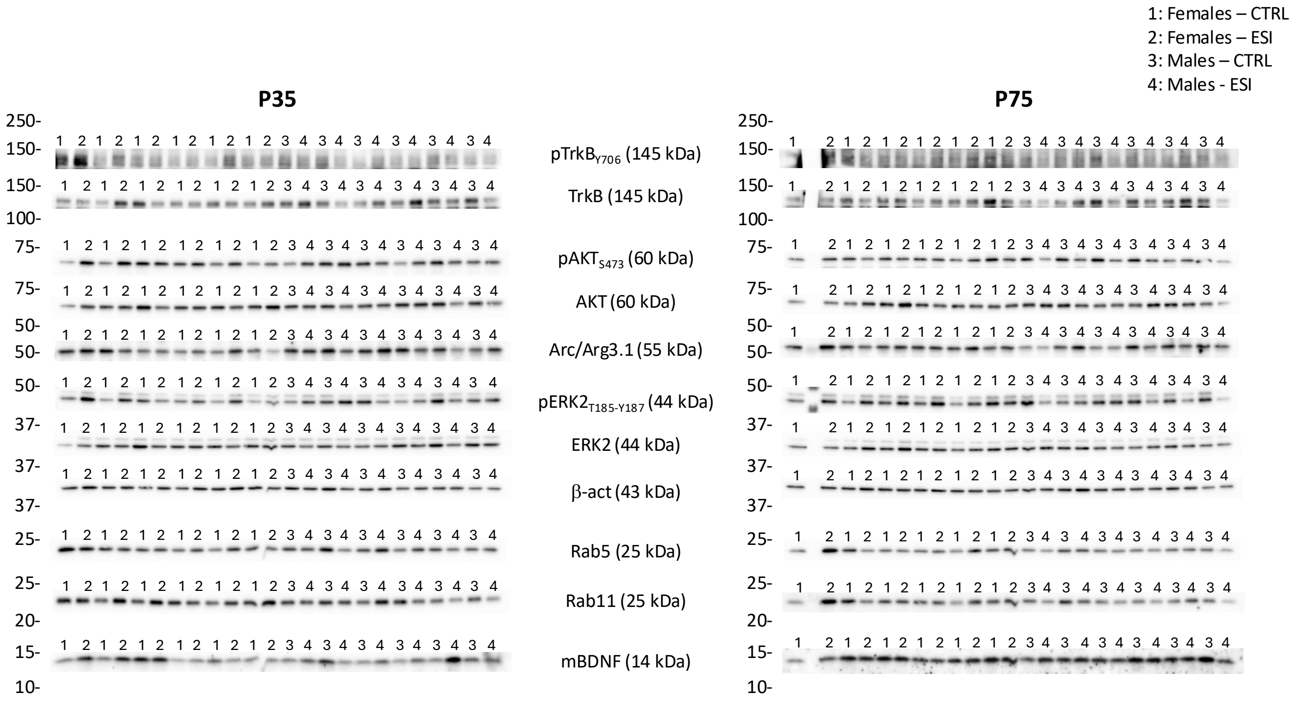

Supplement: Supplementary file 1 — Figure S1: jnc70181‐sup‐0001‐FigureS1.pdf. [file JNC-169-0-s001.pdf]
